# Rational polynomial representation of ribonucleotide reductase activity

**DOI:** 10.1186/1471-2091-6-8

**Published:** 2005-05-06

**Authors:** Tomas Radivoyevitch, Ossama B Kashlan, Barry S Cooperman

**Affiliations:** 1 Epidemiology and Biostatistics, Case Western Reserve University, Cleveland, OH 44106, USA; 2 Medicine, University of Pittsburgh, Pittsburgh, PA 15261, USA; 3 Chemistry, University of Pennsylvania, Philadelphia, PA 19104, USA

## Abstract

**Background:**

Recent data suggest that ribonucleotide reductase (RNR) exists not only as a heterodimer R1_2_R2_2 _of R1_2 _and R2_2 _homodimers, but also as tetramers R1_4_R2_4 _and hexamers R1_6_R2_6_. Recent data also suggest that ATP binds the R1 subunit at a previously undescribed hexamerization site, in addition to its binding to previously described dimerization and tetramerization sites. Thus, the current view is that R1 has four NDP substrate binding possibilities, four dimerization site binding possibilities (dATP, ATP, dGTP, or dTTP), two tetramerization site binding possibilities (dATP or ATP), and one hexamerization site binding possibility (ATP), in addition to possibilities of unbound site states. This large number of internal R1 states implies an even larger number of quaternary states. A mathematical model of RNR activity which explicitly represents the states of R1 currently exists, but it is complicated in several ways: (1) it includes up to six-fold nested sums; (2) it uses different mathematical structures under different substrate-modulator conditions; and (3) it requires root solutions of high order polynomials to determine R1 proportions in mono-, di-, tetra- and hexamer states and thus RNR activity as a function of modulator and total R1 concentrations.

**Results:**

We present four (one for each NDP) rational polynomial models of RNR activity as a function of substrate and reaction rate modifier concentrations. The new models avoid the complications of the earlier model without compromising curve fits to recent data.

**Conclusion:**

Compared to the earlier model of recent data, the new rational polynomial models are simpler, adequately fitting, and likely better suited for biochemical network simulations.

## Background

Ribonucleotide reductase (RNR) is a key component of *de novo *deoxynucleotide (dNTP) metabolism and an important target of cancer therapies [1]. This enzyme, which reduces ribonucleoside diphosphates into corresponding deoxyribonucleoside diphosphates, is exquisitely controlled to properly balance dNTP fluxes in the face of changing scheduled (S phase) and unscheduled (DNA damage/repair) dNTP synthesis demands [2].

**Figure 1:**
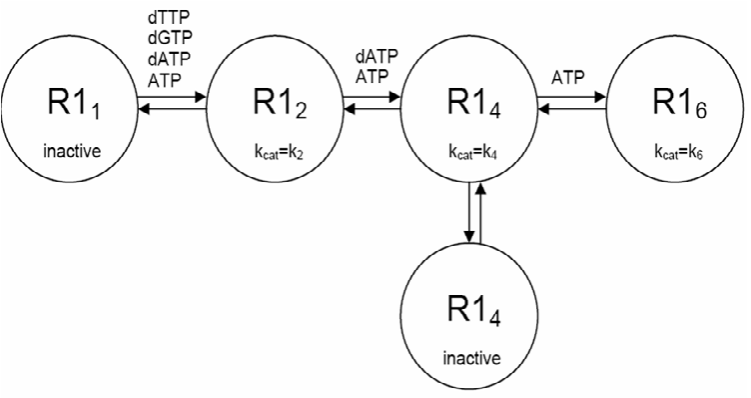
Quaternary states of R1. Modulators of RNR activity listed in this figure bind R1 to create higher order quaternary states. Tetramers exist in an equilibrium between low activity states (see k_4 _in Table 1) and inactive states (k_cat _= 0). Adapted from Scheme 1 in [4,5].

Recent data [3-6] suggest that ribonucleotide reductase (RNR) exists not only as a heterodimer R1_2_R2_2 _of R1_2 _and R2_2 _homodimers [2], but also as a R1_4_R2_4 _tetramer and as a R1_6_R2_6 _hexamer, where hexamer formation is driven by ATP binding to a previously undescribed hexamerization site. Thus, in addition to its four substrate binding possibilities in ADP, GDP, CDP, or UDP, and four dimerization/specificity site binding possibilities in dATP, ATP, dGTP, or dTTP, the current view (Figure 1) is that R1 has two tetramerization/inhibitory site binding possibilities in dATP or ATP, and one hexamerization/activation site binding possibility in ATP, in addition to possibilities of unbound site states. The resulting large number of possible R1 states implies an even larger number of quaternary states, and this leads to a complicated mathematical model of RNR activity [3-6]. This model, although useful for explaining RNR activity data, is not useful for biochemical network simulations because: a) it is unwieldy (including up to six-fold nested sums), b) it uses different mathematical structures under different substrate-modulator conditions, and c) it requires root solutions of high order polynomials to determine R1 proportions in mono-, di-, tetra- and hexamer quaternary states, and thus RNR activity, as a function of modulator and total R1 concentrations. Simpler mathematical reaction rate models of RNR are needed if deoxynucleotide metabolism [7] is to be represented using Systems Biology Markup Language (SBML) [8-10], a standard which requires single algebraic expression reaction rate laws in some applications [11,12]. Based on recent data from Cooperman's group [4-6], such expressions are provided here for RNR.

## Results

Reaction activities are viewed here as weighted sums of enzyme state specific activities multiplied by probabilities of enzymes being in specific states. For example, a Michaelis-Menten reaction rate law is viewed as



where the probability that the enzyme is in a loaded/reactive state (with activity *k*_cat_) is P(EA) and the probability that the enzyme is in an empty/unreactive state (with no activity) is P(E).

The RNR models presented here are based on the following four enzyme state probability assumptions:

1. The probability that a particular R1 subunit is bound to NDP is assumed to be



2. The probability that *L *∈ {*ATP, dATP, dTTP, dGTP*} is also bound to the dimerization/specificity site, conditional on NDP binding, is assumed to be



where, if *L *= *dTTP *and *NDP *= *GDP *for example,



is the probability that dTTP is bound to the dimerization/specificity site given that GDP is bound to the substrate site; the probability of an empty dimerization/specificity site is thus



3. The probability that the tetramerization site is either empty, occupied by ATP, or occupied by dATP, is assumed to be, respectively,







4. Finally, the probability that the hexamerization site is occupied by ATP is assumed to be



The subscripts *a *(activation), *i *(inactivation), and *s *(specificity) on the binding constants correspond to *h *(hexamerization), *t *(tetramerization), and *d *(dimerization) subscripts on *P*, respectively. That parameter values differ depending upon which NDP substrate is bound to the active site (see Table 1) is indicated by the conditional probabilities.

**Table 1:** Parameter estimates of the reductase models. Fits to data are as shown in Figure 2.

substrate	K_sdATP_	K_sATP_	K_sdTTP_	K_sdGTP_	K_idATP_	K_iATP_	K_aATP_	k_2_	k_2dA_	k_2A_	k_2e_	k_4_	k_6_
ADP	2	200	2.4^b^	0.5	1.25	300	2000^d^	0.21				0.03	0.16
GDP	1	100	0.5	2	2	190	2400	0.28^a^				0.04	0.19
CDP	2	70	1.55^b^	2^c^	1.5	600	1400		0.25^a^	0.29^a^	0.08		0.32
UDP	1	100	0.7^b^	2^c^	0.5	200	800		0.26	0.26			0.26^a^

Binding constants in μM, rate constants in 1/s.

^a^fixed values taken directly from table 5 of [5].

^b^using Eq. 10, these were adjusted to yield fluxes of 12.5, 12.5, 20 and 5 (uM/min) for ADP, GDP, CDP and UDP, respectively, under assumptions of E_0 _= 16 μM, ADP = 430 μM, GDP = 110 μM, CDP = 55 μM, UDP = 170 μM, K_mADP _= 12 μM, K_mGDP _= 4.9 μM, K_mCDP _= 2 μM, K_mUDP _= 6.4 μM, ATP = 1450 μM, dATP = 10.5 μM, dGTP = 7.3 μM, dTTP = 30 μM and the remaining parameter values in Table 1, see [7].

^c^no data, thus, these can be assumed to have any value between .5 and 2; a default value of 2 was carried down from GDP.

^d^no data, 2000 is based on the other rows.

Previous work [3-6] has shown that the dimer and hexamer states are active, that the tetramer state is slightly active for ADP and GDP and is otherwise inactive, that dimer state activity for CDP and UDP exists when ATP or dATP is bound to the dimerization/specificity site, and that an empty dimerization/specificity site still permits the formation of some dimer with CDP reductase activity, see Table 5 of [5]. Thus, based on the enzyme state probabilities given above, for *k*_cat _implicitly defined through



we propose the following expressions:

ADP reduction



GDP reduction



CDP reduction



UDP reduction



**Figure 2:**
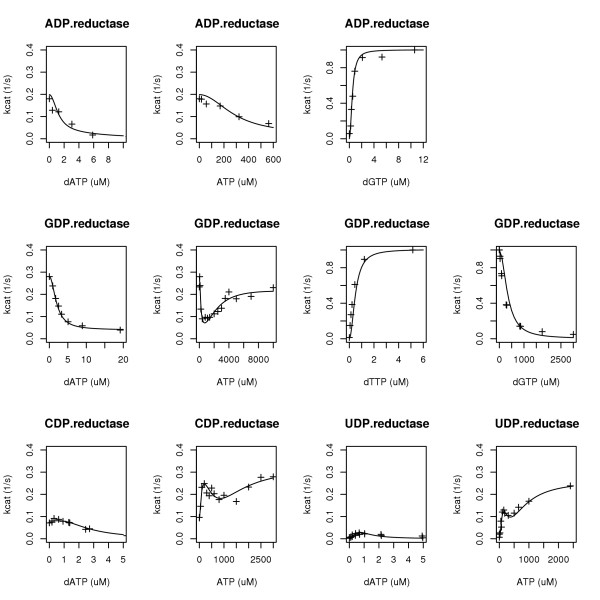
Data from [4-6] and corresponding curve fits (Table 1) of the RNR activity models. In these plots, from left to right, for ADP reduction dGTP was 2.1 uM or variable, for GDP reduction dTTP was 100 uM, 300 uM, variable, or 85 uM, and for pyrimidines specificity site binding concentrations were as shown. In all cases NDP and R2 were at saturating levels.

In these equations, for ADP and GDP, the first factor is the probability that the dimer site is occupied, and for CDP and UDP, the first factor is the expectation of k_cat _conditional on R1 being in a dimer state (i.e. having an empty tetramerization site). In the ADP and GDP models, the second factor is the conditional expectation of k_cat _given that the dimerization site is occupied: the first term of this second factor has in its numerator the statement that k_cat _= k_2 _if the tetramerization site is empty, or k_cat _= k_4 _if it is occupied by either dATP or ATP, and the second term states that k_cat _= k_6 _if the hexamerization site is occupied by ATP. For the CDP and UDP models, the first term of the second factor is the probability of an empty tetramerization site (the event that the corresponding first factor was conditioned on), and the second term states that if the ATP concentration is high enough that the hexamerization/second term dominates the tetramerization/first term whilst the first factor approaches k_2A_, k_6 _is the overall k_cat_. This rationale served as our model selection guide. Importantly, the models fitted recent data [3-6] very well, see Figure 2 and Table 1.

## Discussion

In general, when an integrated system is engineered from component subsystems, the behavior of the overall system depends on component input-output specifications more so than the details of component implementations. By analogy, when enzymological data are applied to biochemical network modeling, rather than the elucidation of reaction mechanisms, it can be expected that the reaction surfaces themselves (i.e. the enzyme's input-output characteristics) determine network behavior more so than the details of how such surfaces are represented. Thus, for applications to systems biology, large confidence intervals (CI) in the model parameter estimates of Table 1 (not shown) are not a problem because only goodness-of-fit (Fig. 2) really matters; this claim assumes an operating range within the data range, since similarly fitting models often veer apart when used in extrapolations. If reaction mechanism inferences were instead being sought, the large CI in the model parameter estimates would have been a problem, e.g. the squared terms in the model suggest cooperative binding, but this choice provides only slightly better curve fits compared to linear terms (not shown), so cooperative binding cannot be inferred from this model.

**Figure 3:**
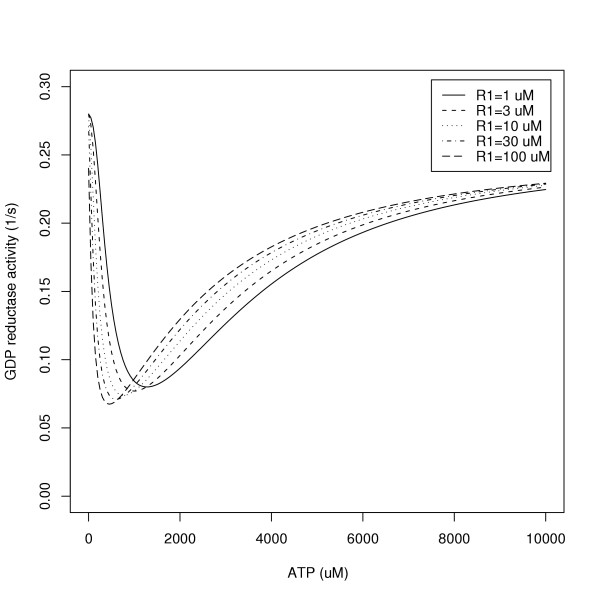
GDP reductase as a function of ATP concentration at various R1 concentrations, predicted using the earlier model [3-6].

In the RNR model presented here, the proportion of R1 units existing in monomer, dimer, tetramer, or hexamer states, and thus the RNR activity per unit enzyme, depends on site binding occupancies but does not depend on the total R1 concentration. In the more complicated previous model [3-6], higher total R1 concentrations favor higher order quaternary states. The degree to which this is so is illustrated by plots of predicted GDP reductase activity as a function of ATP concentration at various R1 concentrations (Figure 3). Consistent with the formation of higher order quaternary R1 states, these plots contract to the left as the total R1 concentrations increase from 1 μM to 100 μM. In future work, the model given here will be altered to capture such trends without losing its simplicity; the total R1 concentration will enter such a model not only as a linear modulator of the reaction surface amplitude (i.e. E_0 _in Eq. 10), but also as a modifier of reaction surface shape parameters, e.g. *K*_aATP _will be replaced by a decreasing function of R1.

## Conclusion

We identified a rational polynomial model of RNR activity that has single algebraic expressions for each reductase reaction rate law. The expressions provide reasonably good fits (Fig. 2) to recent data [3-6]. Compared to previous reaction rate expressions for this data [3-6], the new expressions are simpler and thus better suited for biochemical network simulations, particularly those constrained to use enzyme reaction rate laws defined as single algebraic expressions [11,12].

## Methods

The parameter estimates shown in Table 1 were obtained through a trial-and-error iterative process of nonlinear least squares curve fitting under various, convergence enabling, parameter fixations (i.e. profile searches). In the end, the curve fits were those of Figure 2 with corresponding parameter estimates in Table 1; large 95% confidence intervals (not shown) allowed rounding of the parameter estimates to somewhat arbitrary choices. Non-linear least squares parameter estimations were performed using the optimization method of Nelder and Mead [13] and the statistical computing environment R [14]. All parameters were estimated as e^c ^to assure positive values. For additional details, R scripts are available with the data as supplementary material [15].

## List of abbreviations

RNR = ribonucleotide reductase; dNTP = deoxynucleoside tripshospate; dNDP = deoxynucleoside dipshospate; *a *= activation; *i *= inactivation; *s *= specificity; *h = *hexamerization; *t *= tetramerization; *d *= dimerization; CI = confidence interval; SBML = Systems Biology Markup Language.

## Authors' contributions

TR performed the curve fits to the data and explored various model choices.

OBK and BSC provided the original model, its simulations (Figure 3) and the data.
